# Variants of *SLC39A4* cause acrodermatitis enteropathica in Tibetan, Yi, and Han families in Sichuan region of southwestern China: a case report series

**DOI:** 10.3389/fmed.2024.1399511

**Published:** 2024-05-20

**Authors:** Zhongtao Li, Sheng Wang

**Affiliations:** ^1^Department of Dermatology, West China Hospital, Sichuan University, Chengdu, China; ^2^Laboratory of Dermatology, Clinical Institute of Inflammation and Immunology (CIII), Frontiers Science Center for Disease-related Molecular Network, West China Hospital, Sichuan University, Chengdu, China

**Keywords:** acrodermatitis enteropathica, normal serum zinc, pustule, Tibetan, *SLC39A4*

## Abstract

Acrodermatitis enteropathica (AE, OMIM 201100) is a rare autosomal recessive dermatosis characterized by periorificial dermatitis, diarrhea, alopecia, and hypozincaemia due to pathogenic variants of *SLC39A4*. Herein, we present a case series describing four unrelated patients with AE from Han, Yi, and Tibetan ethnicities in Sichuan region of southwestern China, speculate the hotspot variants of *SLC39A4* causing AE in Sichuan region and highlight physicians should be alerted to unusual presentations of AE, such as the absence of hypozincaemia and the presence of acne-like lesions. Serum alkaline phosphatase and genetic testing should be considered to accurately evaluate the zinc deficiency in human body and help make the correct diagnosis.

## 1 Introduction

Acrodermatitis enteropathica (AE, OMIM 201100) is a rare autosomal recessive skin disease characterized by periorificial dermatitis, diarrhea, and alopecia. It is usually associated with hypozincaemia due to the defect in ZIP4 zinc transporter encoded by *SLC39A4*. Two Tibetan patients with AE from Sichuan region of southwestern China had been previously reported by our group (1, 2). Herein, we added another four patients from Han, Yi, and Tibetan families in Sichuan region and reported the unusual presentations in AE.

## 2 Case reports

### 2.1 Case 1

**Figure 1:**
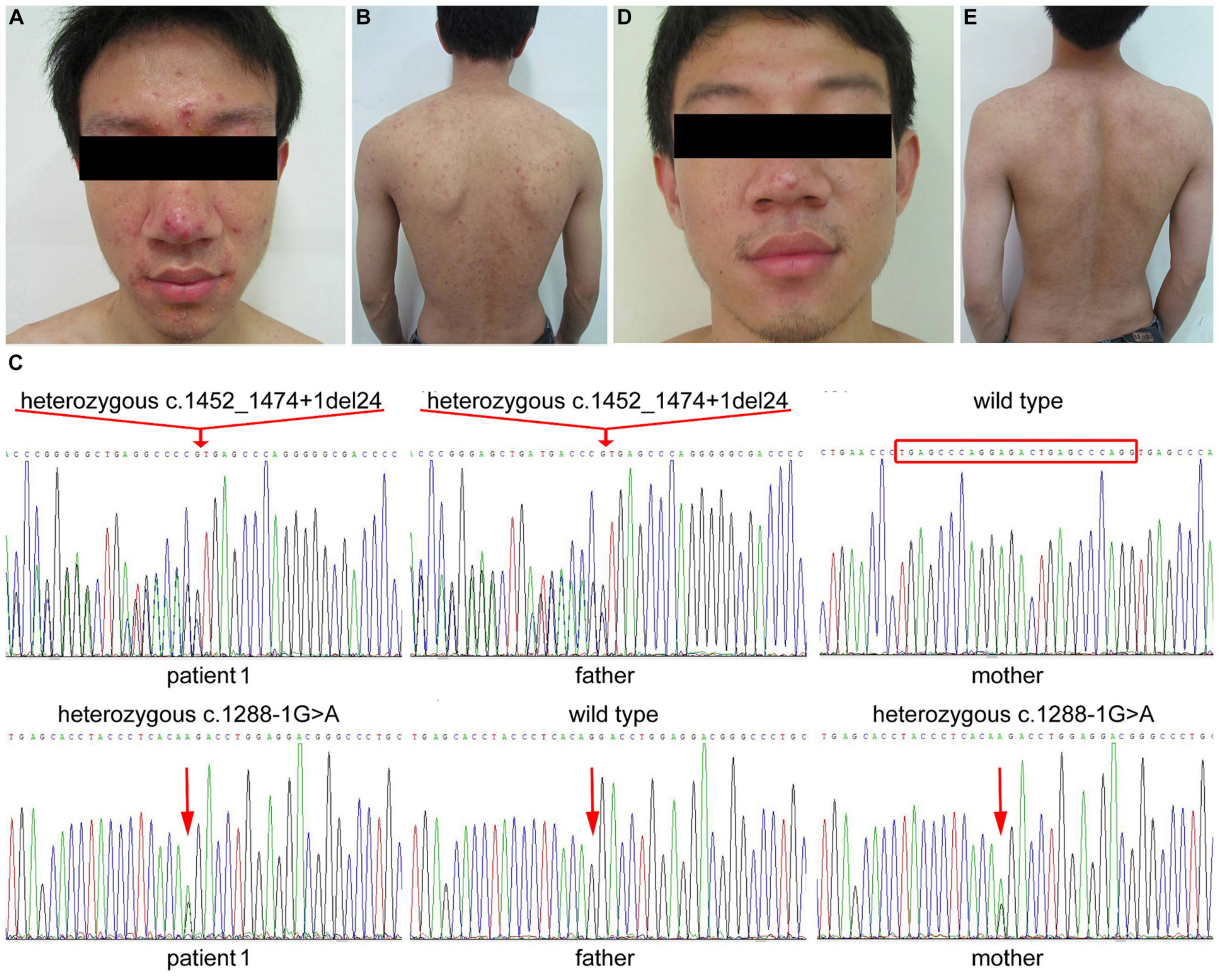
Clinical manifestations and variants of *SLC39A4* in patient 1 with AE. **(A,B)** Generalized pustules on the face and trunk prior to the treatment. **(C)** Heterozygous deletion of TGAGCCCAGGAGACTGAGCCCAGG in exon 9 and the adjoining intron 9 in patient 1 and his father. Heterozygous splicing variant c.1288-1G > A in intron 7 in patient 1 and his mother. **(D,E)** Marked relief of the lesions after 7 weeks of zinc supplementation.

An 18-year-old man, born to non-consanguineous, healthy Han parents, presented with papules and pustules on the face and extremities since the age of 4 years. Physical examination revealed erythema, papules, pustules, crusts and scars on the face, trunk and periorificial region (Figures 1A,B). Diarrhea and hair loss were absent. He was repeatedly diagnosed with “acne” in local hospitals, but the anti-acne treatment with minocycline achieved no improvement. The serum zinc level was 103.8 μmol/L (normal 76.5–170.0 μmol/L), while the serum alkaline phosphatase level was only 41 IU/L (lower than the normal range of 45–125 IU/L). Sanger sequencing was performed using the genomic DNA of the patient and his parents. The patient was found to carry a 24-bp heterozygous deletion in exon 9 and intron 9 (c.1452_1474 + 1del TGAGCCCAGGAGACTGAGCCCAGG) and a heterozygous splicing variant in intron 7 (c.1288-1G > A) of *SLC39A4* (RefSeq NM_130849.4). His father and mother were found to be heterozygous for c.1452_1474 + 1del TGAGCCCAGGAGACTGAGCCCAGG and c.1288-1G > A, respectively (Figure 1C). Thus, the diagnosis of AE was established. After 7 weeks of continuous treatment with a single oral zinc sulfate (50 mg three times daily), his skin lesions almost completely disappeared (Figures 1D,E) and the serum level of alkaline phosphatase increased to 90 IU/L.

### 2.2 Case 2

**Figure 2:**
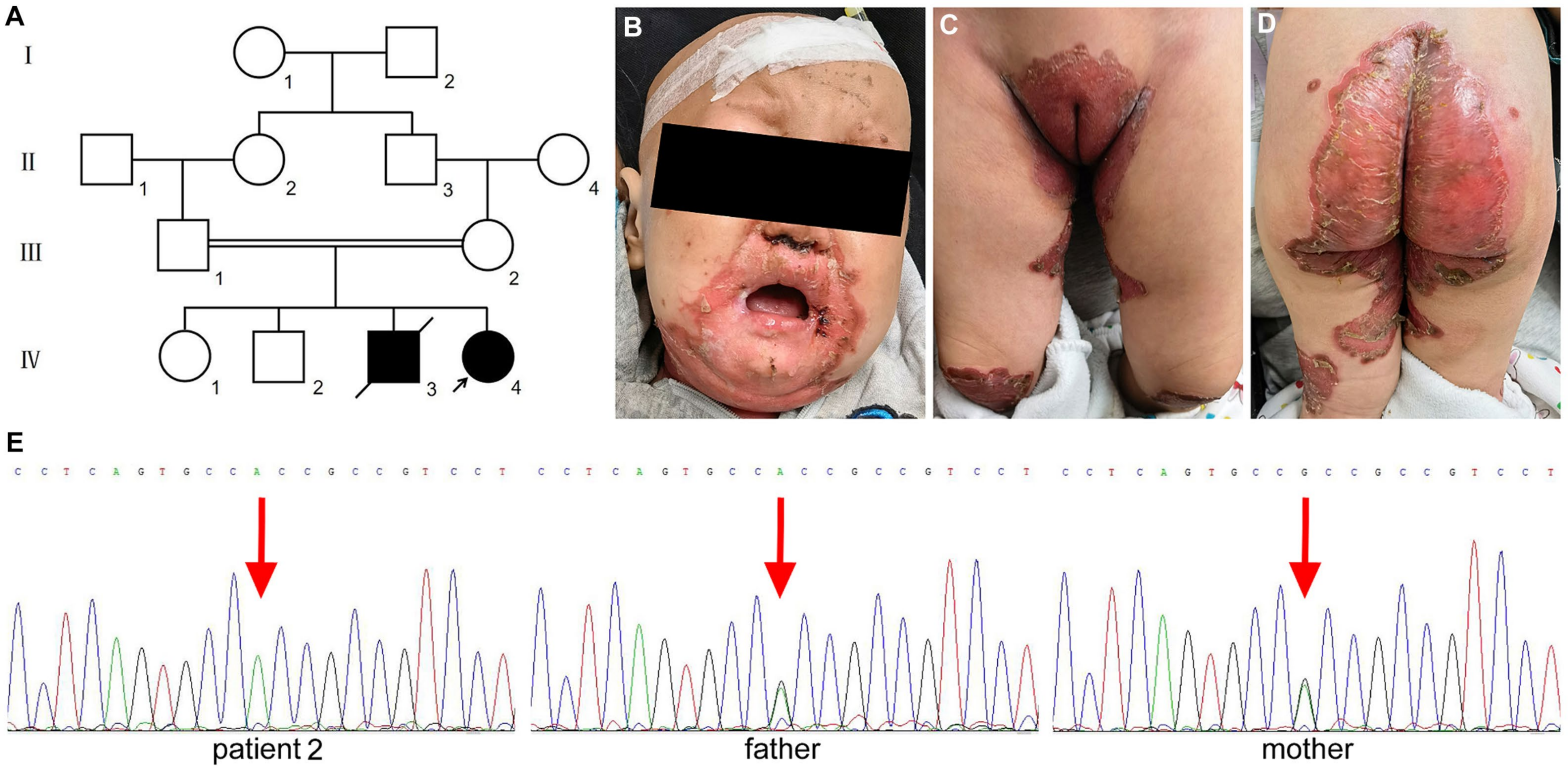
Clinical findings and the variant of *SLC39A4* in patient 2 with AE. **(A)** Pedigree of the Chinese Yi family of patient 2. **(B–D)** Erythema and crusted lesions in the periorificial region and extensor surface of the knees. **(E)** Homozygous missense variant of c.295G > A in exon 2 of patient 2 and heterozygous variant for her parents.

The patient was a girl aged 1 year and 10 months, the fourth child of second-degree consanguineous parents with Yi ethnicity (Figure 2A; IV: 4). Her parents reported erythema and crusts appeared on her periorificial areas accompanied with alopecia and chronic diarrhea since 4 months after birth. Her older brother had a history of similar lesions and alopecia, but had already died at the age of 3 years (Figure 2A; IV: 3). Physical examination revealed well-demarcated erythema and crusts on her hands, feet, extensor surface of the knees and in the perioral, periocular, perigenital, and perianal regions (Figures 2B–D). Her serum zinc level was 0.13 mg/L (normal 0.76–1.50 mg/L) and the serum alkaline phosphatase level was only 22 IU/L (normal 128–432 IU/L). Sanger sequencing analysis showed the patient carried a homozygous c.295G > A (p.Ala99Thr) in exon 2 of *SLC39A4* (RefSeq NM_130849.4) (Figure 2E). Considering the diagnosis of AE, zinc supplementation at 3 mg per kg-body-weight was started, which led to complete remission after 2 weeks.

### 2.3 Case 3

**Figure 3:**
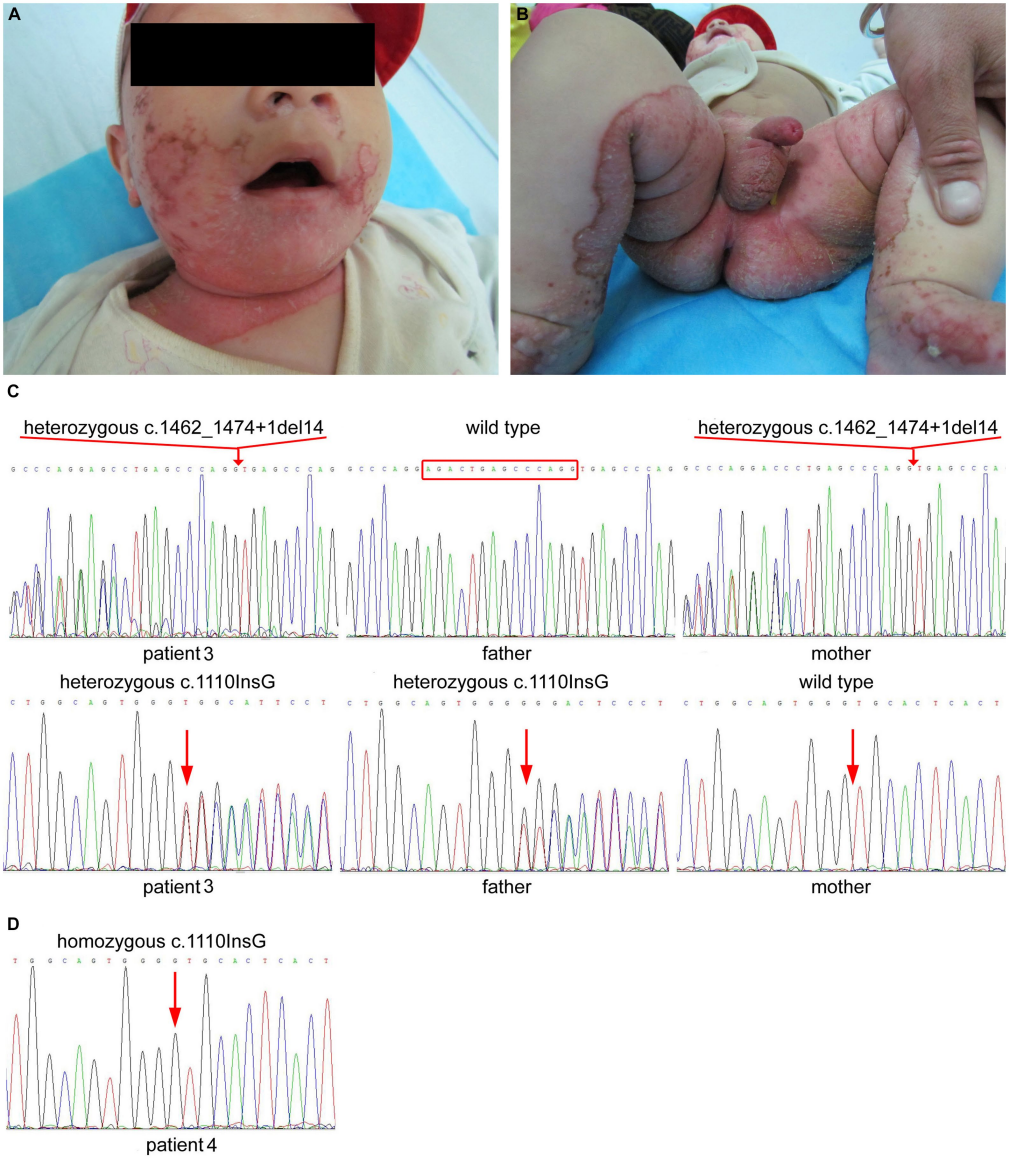
**(A,B)** Clinical findings of patient 3 showed well-demarcated erythema and crusted lesions in the neck, feet, periorificial region, and flexor surface of the lower legs. **(C)** Heterozygous deletion of AGACTGAGCCCAGG in exon 9 and the adjoining intron 9 of *SLC39A4* in patient 3 and his mother. Heterozygous frame-shift variant of c.1110insG in exon 6 of *SLC39A4* in patient 3 and his father. **(D)** Homozygous frame-shift variant of c.1110insG in exon 6 of *SLC39A4* in patient 4.

An 8-month-old Tibetan boy was referred to our department for evaluation of exanthema on the face and anogenital regions, which had developed approximately 4 months after birth. He had diarrhea, hair loss and irritability. There was no history of any consanguineous marriage in the family. Examination revealed well-demarcated erythema and crusts on the neck, flexor side of the lower extremities and in the perioral, periocular, perigenital, and perianal regions (Figures 3A,B). Laboratory data were within normal limits except for reduced serum zinc 28.44 μmol/L (normal 33.5–84.54 μmol/L). The detection of serum alkaline phosphatase was not performed. Sanger sequencing of *SLC39A4* (RefSeq NM_130849.4) revealed that the patient was compound heterozygous for maternal c.1462_1474 + 1del AGACTGAGCCCAGG in exon 9 and intron 9 and paternal c.1110insG in exon 6 (Figure 3C). Both variants have been reported to be pathogenic (1, 3). The patient showed marked improvement after 2 weeks of oral zinc supplements.

### 2.4 Case 4

A 10-year-old Tibetan girl presented with eczematous lesions in the perioral, perigenital, and perianal regions accompanied with diarrhea since 7 months after weaning. No alopecia was observed. She had been diagnosed with AE and the skin lesions had been almost disappeared with oral zinc supplements when she came to our department. The patient’s serum zinc level and serum alkaline phosphatase were not detected. No consanguinity was reported in the family. Sanger sequencing of all exons and their flanking intronic regions of *SLC39A4* (RefSeq NM_130849.4) identified a homozygous c.1110insG variant in the patient (Figure 3D).

## 3 Discussion

*SLC39A4* have been found to be responsible for the development of AE (1). Pathogenic variants of *SLC39A4* would impair the zinc absorption of the ZIP4 transporter in the small intestine, leading to the zinc deficiency, further resulting in various clinical manifestations, such as skin dermatitis, alopecia, diarrhea, growth retardation, and neuropsychiatric symptoms (4). To date, 14 cases with *SLC39A4* variants have been found in AE patients from China in the literature, including four cases described here (Table 1) (1–10). Among them, more than half (*n* = 8, 57.14%) were from Sichuan region of southwestern China, indicating it may be one of the high AE incidence areas. In addition, not all of them simultaneously have the classic triad of skin dermatitis, alopecia, and diarrhea. Growth retardation and neuropsychiatric symptoms were found in a few patients with AE.

**Table 1:** Summary of cases of AE with the *SLC39A4* variant from China.

Case	Year	Authors	Sex	Age of onset	Origin	History of consanguinity	Family members have similar symptoms	Clinical manifestations				Serum zinc level	Serum alkaline phosphatase level	*SLC39A4* variants
								Skin lesions	Alopecia	Diarrhea	Other symptoms			
1	2008	Wang et al. (1)	F	3 months	Sichuan (Tibetan)	Yes	Older brother	Atypical (generalized pustules)	Yes	Yes	No	Low	Low	Homozygous for c.1462_1474 + 1del AGACTGAGCCCAGG (exon 9 and intron 9)
2	2010	Li et al. (5)	F	5 months	Jiangsu	Yes	No	Typical	No	Yes	No	Low	Not detected	Homozygous for c.1115 T > G (exon 6)
3	2014	Gao et al. (6)	M	Infancy	Jiangsu	No	No	Typical	No	Yes	Growth retardation	Low	Low	Compound heterozygous for c.831G > A (exon 5) and c.1617delA (exon10)
4	2016	Zhou et al. (2)	F	Several months after birth	Sichuan (Tibetan)	Yes	Older brother and sister	Typical	Yes	Yes	No	Low	Not detected	Homozygous for c.1462_1474 + 1del AGACTGAGCCCAGG (exon 9 and intron 9)
5	2017	Mou et al. (3)	M	Several days after birth	Sichuan (Han)	Yes	No	Typical	Yes	Yes	Anorexia, apathia	Low	Not detected	Compound heterozygous for c.1110InsG (exon 6) and c.958C > T (exon 5)
6	2019	Wu et al. (7)	F	2 months	Guangdong	Yes	Two brothers and two sisters	Typical	Yes	No	No	Normal	Low	Homozygous for c.106C > T (exon 1)
7	2020	Zhou et al. (8)	M	3 days	Guangdong	No	No	Typical	No	Yes	No	Low	Not detected	Homozygous for c.948delC (exon 5)
8	2020	Zhong et al. (4)	M	12 months	Guangdong	No	Twin brother	Typical	No	No	Apathia	Normal	Normal	Compound heterozygous for c.926G > T (exon 5) and c.976 + 2 T > A (intron 5)
9	2022	Li et al. (9)	F	3 months	Hunan	No	Younger brother	Typical	Yes	No	No	Normal	Not detected	Homozygous for c.1456delG (exon9)
10	2022	Hua et al. (10)	F	1 year	Sichuan (Han)	No	No	Typical	Yes	No	No	Low	Not detected	Compound heterozygous for c.1466dupT (exon 9) and c.295G > A (exon 2)
11	2024	Present patient 1	M	4 years	Sichuan (Han)	No	No	Atypical (acne-like pustules)	No	No	No	Normal	Low	Compound heterozygous for c.1452_1474 + 1del TGAGCCCAGGAGACTGAGCCCAGG (exon 9 and intron 9) and c.1288-1G > A (intron 7)
12	2024	Present patient 2	F	4 months	Sichuan (Yi)	Yes	Older brother	Typical	Yes	Yes	No	Low	Low	Homozygous for c.295G > A (exon 2)
13	2024	Present patient 3	M	4 months	Sichuan (Tibetan)	No	No	Typical	Yes	Yes	Irritability	Low	Not detected	Compound heterozygous for c.1462_1474 + 1delAGACTGAGCCCAGG (exon 9 and intron 9) and c.1110insG (exon 6)
14	2024	Present patient 4	F	7 months	Sichuan (Tibetan)	No	No	Typical	No	Yes	No	Not detected	Not detected	Homozygous for c.1110insG (exon 6)

AE, Acrodermatitis enteropathica; F, Female; M, Male.

A total of 15 *SLC39A4* variants have been reported in AE patients from China, almost half of which, seven variants, were found in Sichuan region (Table 1). Unlike other areas, some variants, including c.295G > A, c.1110insG and c.1462_1474 + 1del AGACTGAGCCCAGG, were repeatedly identified in Sichuan region. In particular, the c.1462_1474 + 1del AGACTGAGCCCAGG variant was exclusively identified in Tibetan ethnicity. Given the enclosed environment and prevalence of consanguineous marriage in this region, besides hotspot variants, we consider the founder effect is likely to exist.

Acrodermatitis enteropathica is typically manifested by periorificial dermatitis and usually associated with hypozincaemia. However, a few patients could exhibit some atypical lesions, such as generalized pustules and blisters mimicking infectious diseases, or plaques resembling psoriasis and palmoplantar keratoderma (1, 11, 12). Moreover, serum zinc level could be normal in a minority of patients with AE (7). As in this study, the skin lesions of patient 1 presented with erythema, papules, pustules, and scars on the face and trunk, which were so much like acne. In addition, his serum zinc level was normal but the serum alkaline phosphatase level was low. The acne-like lesions improved obviously and serum alkaline phosphatase level increased to the normal range after administration with a single regimen of zinc sulfate. Under this condition, patients with low serum alkaline phosphatase level presenting acne-like lesions should raise a high suspicious of atypical AE. Detection of zinc-dependent metalloenzyme serum alkaline phosphatase, a more sensitive and precise parameter for the assessment of zinc deficiency, is very essential to help make a correct diagnosis.

## 4 Conclusion

Overall, in this study, a total of five variants of *SLC39A4*, including two unreported before, was identified in four unrelated patients with AE from three different ethnicities in Sichuan region of southwestern China. The three recurrent variants, c.295G > A, c.1110insG, and c.1462_1474 + 1del AGACTGAGCCCAGG, seem to be hotspot variants of *SLC39A4* in patients with AE from Sichuan region. Of note, the c.1462_1474 + 1del AGACTGAGCCCAGG variant was exclusively identified in Tibetan ethnicity, implying a founder effect is likely to exist. Sichuan region of southwestern China may be one of the high AE incidence areas. Unusual acne-like lesions developed in a Han patient with a normal serum zinc level, which highlights the diversity of clinical manifestations of AE. Serum alkaline phosphatase and genetic tests are necessary for evaluating the zinc deficiency in human body and helping make the correct diagnosis. In this study, some limitations should not be neglected, especially the small sample size. Therefore, more works are needed to verify our findings based on larger sample size in the future.

## Data availability statement

The raw data supporting the conclusions of this article will be made available by the authors, without undue reservation.

## Ethics statement

The studies involving humans were approved by the ethics committee of West China Hospital, Sichuan University. The studies were conducted in accordance with the local legislation and institutional requirements. Written informed consent for participation in this study was provided by the participants’ legal guardians/next of kin. Written informed consent was obtained from the individual(s), and minor(s)’ legal guardian/next of kin, for the publication of any potentially identifiable images or data included in this article.

## Author contributions

ZL: Data curation, Formal Analysis, Methodology, Writing – original draft. SW: Conceptualization, Resources, Supervision, Validation, Writing – review & editing.

## References

[1] Wang S Xue L Guo ZP Wang L Yang Y. A novel SLC39A4 gene mutation in the family of an acrodermatitis enteropathica patient with an unusual presentation. Br J Dermatol. (2008) 159:976–8. doi: 10.1111/j.1365-2133.2008.08777.x, PMID: 18684156

[2] Zhou XY Chen XJ Wang S Xue J Liu W Wang Q. One recurrent homozygous mutation of SLC39A4 in a girl with acrodermatitis enteropathica from southwestern China. Int J Dermatol. (2016) 55:223–5. doi: 10.1111/ijd.12442, PMID: 24962159

[3] Mu Y Zhang Z Yang P Yang H Liu Y Liu L. Analysis of SLC39A4 gene mutation in a patient with acrodermatitis enteropathica. Zhonghua Yi Xue Yi Chuan Xue Za Zhi. (2017) 34:387–9. doi: 10.3760/cma.j.issn.1003-9406.2017.03.016, PMID: 28604961

[4] Zhong W Yang C Zhu L Huang YQ Chen YF. Analysis of the relationship between the mutation site of the SLC39A4 gene and acrodermatitis enteropathica by reporting a rare Chinese twin: a case report and review of the literature. BMC Pediatr. (2020) 20:34. doi: 10.1186/s12887-020-1942-4, PMID: 31987033 PMC6983971

[5] Li CR Yan SM Shen DB Li Q Shao JP Xue CY. One novel homozygous mutation of SLC39A4 gene in a Chinese patient with acrodermatitis enteropathica. Arch Dermatol Res. (2010) 302:315–7. doi: 10.1007/s00403-010-1047-2 20300938

[6] Gao Y Li X Huang S You SH Fan ZN. A study of SLC39A4 gene mutation responsible for acrodermatitis enteropathica. Acta Univ Med Nanjing. (2014) 34:660–3. doi: 10.7655/NYDXBNS20140525

[7] Wu F Zhang Y Shi X Lu P Yang C Man MQ. Novel nonsense mutation of the SLC39A4 gene in a family with atypical acrodermatitis enteropathica. Clin Exp Dermatol. (2019) 44:933–6. doi: 10.1111/ced.13964, PMID: 30980548

[8] Zhou X Xue R Luo Q Zhang SQ. A study of SLC39A4 gene mutation in acrodermatitis enteropathica. Chin J Dermatovenereol. (2020) 34:739–42. doi: 10.13735/j.cjdv.1001-7089.201909054

[9] Li KY Tang JP Shu Y Qiu SZ Wang YW Wen R. Recurrent systemic sporadic rash for 10 years in a girl aged 11 years. Zhongguo Dang Dai Er Ke Za Zhi. (2022) 24:1047–52. doi: 10.7499/j.issn.1008-8830.2204123, PMID: 36111725 PMC9495228

[10] Hua W Zou J Zhuang Y Zhou T. Case report: Acrodermatitis enteropathica result from a novel SLC39A4 gene mutation. Front Pediatr. (2022) 10:972030. doi: 10.3389/fped.2022.972030, PMID: 36479285 PMC9720256

[11] Iyengar S Chambers C Sharon VR. Bullous acrodermatitis enteropathica: case report of a unique clinical presentation and review of the literature. Dermatol Online J. (2015) 21:13030. doi: 10.5070/D3214026272 25933075

[12] Behera B Mohapatra L Sahu B Patnaik M. A case of acrodermatitis enteropathica mimicking mutilating palmoplantar keratoderma. Indian J Dermatol. (2022) 67:314. doi: 10.4103/ijd.IJD_112_17 PMC9644794 36386094

